# The operationalization of self-identity in reasoned action models: a systematic review of self-identity operationalizations in three decades of research

**DOI:** 10.1080/21642850.2020.1852086

**Published:** 2021-01-28

**Authors:** Marwin H. M. Snippe, Gjalt-Jorn Ygram Peters, Gerjo Kok

**Affiliations:** a Work and Social Psychology, Maastricht University, Maastricht, the Netherlands; b Methodology and Statistics, Open University, Heerlen, the Netherlands

**Keywords:** Theory of planned behavior, reasoned action, self-identity, systematic review

## Abstract

**Background:**

Self-identity has frequently been proposed as a useful addition to the Theory of Planned Behavior; yet Fishbein and Ajzen decided to not include self-identity when they published that theory’s successor, the Reasoned Action Approach. One of their reasons for exclusion is the lack of clear conceptual independence, as manifested in self-identity operationalizations that often conflate the construct with attitudinal or normative measures. Therefore, establishing whether self-identity has added value in the Reasoned Action Approach first requires synthesis of the used operationalisations to develop an operationalization that captures self-identity but not attitude and perceived norm.

**Method:**

In this systematic review we identified 153 articles through the PsycINFO database and descendency approach using Google Scholar. In total, 342 of the operationalisations of self-identity were identified in studies operationalizing it as a potential Reasoned Action Approach extension.

**Results and conclusions:**

After analyzing the full item pool to eliminate duplicates and items that did not measure selfidentity, (also) measured attitudes or norms, did not allow tailoring formulations to target, action, context and time, were not applicable to a wide variety of behaviors, or were ambiguous, seven prototypical items remained. These items lend themselves well for further psychometric study to establish the conceptual independence of self-identity from other Reasoned Action Approach constructs such as attitude and perceived norms.

## Introduction

The Reasoned Action Approach (RAA) and its predecessors, the Theory of Planned Behavior (TPB) and Theory of Reasoned Action (TRA), are frequently applied to explain health behavior. The wide application of RAA may in part be a consequence of the flexibility afforded by the TPB, the original formulation of which was explicitly ‘open to the inclusion of additional predictors’ (Ajzen, 1991, p. 199). Many researchers have suggested potential additions to the theory’s three behavioral determinants: attitude, representing perceived consequences of a behavior and their desirability; perceived norms, representing perceived approval or disapproval and perceived behavior of social referents; and perceived behavioral control, representing the perceived capability to perform a behavior and the control over the performance (Fishbein & Ajzen, 2010, p. 154).

This practice of adding predictors to the model fits with the history of Reasoned Action models. In 1967, TRA explained intention with attitude and subjective norm. In 1991 Ajzen, introduced the TPB, adding perceived behavioral control. In the RAA, attitude distinguished instrumental and affective expectations and evaluations, subjective norm was replaced by perceived norms that also included descriptive norms; and perceived behavioral control was delineated into autonomy and capacity. After each reformulation, new predictors were added and the explained variance of intention was increased. For example, the inclusion of descriptive norms can add an additional 5% after controlling for attitude, subjective norms and perceived behavioral control (Rivis & Sheeran, 2003). A second example of the increase in explained variance is a comparison between a review of TPB in 1996 and a meta-analysis of RAA published in 2016. In the TPB review, an average of 41% of intention’s variance was explained (Godin & Kok, 1996); and in the RAA review, 58% of intention was explained (McEachan et al., 2016). Given this openness to including additional constructs on the condition that there is evidence that those inclusions lead to better prediction of intention, it is no surprise that research has focused on adding predictors. One of these potential predictors is self-identity (various definitions will be discussed at the end of the introduction), which has been shown to increase explained variance with 6% (for a review, see Rise, Sheeran, & Hukkelberg, 2010).

However, adding more explained variance to the prediction of intention is not the only criterion for inclusion in the RAA. Fishbein and Ajzen (2010) reviewed the evidence for including self-identity and rejected inclusion for two reasons. First, they argued that the items that had thus far been used to measure self-identity instead operationalize aspects of other RAA constructs, specifically importance, descriptive norms, actual behavior, or current behavior. Therefore, they argued that the operationalization of self-identity is not conceptually independent from the existing RAA constructs, and that sufficiently extensive operationalization of those other constructs would render self-identity redundant.

Fishbein and Ajzen’s second argument is less straightforward and does again concern explained variance. Their argument is based on the RAA’s sufficiency assumption: ‘intentions […] should be predictable from attitude toward the behavior, perceived norm, and perceived behavioral control. Consideration of additional variables should improve prediction of either intention or behavior’ (Fishbein & Ajzen, 2010, p. 281). They then report that past behavior does improve the prediction of intention over the other three RAA variables, and then argue that

one interpretation of the finding that the effect of past behavior on intentions is not fully mediated by attitudes, perceived norms, and perceived control is that the sufficiency assumption is invalid. In other words, intentions may be determined not only by attitudes, norms, and perceived control but also by one or more additional variables, and these additional variables are captured, at least in part, by measures of past behavior. (Fishbein & Ajzen, 2010, p. 289–290)

Based on this reasoning, they use the data of the meta-analysis of Rise et al. (2010) to show that self-identity increases explained variance in intention from 34% (with only attitude, perceived norms, and perceived behavioral control) to 46% (a 12 percentage point increase). However, adding past behavior increases the explained variance further to 53% (a 7 percentage point increase). They then argue that ‘the direct effect of past behavior on intentions cannot be attributed to a failure to include self-identity’ (Fishbein & Ajzen, 2010, p. 294). They provide no justification for this implicit rejection of other potential sources of variance in intention that may be mediated by determinants of intention.

The present article will focus on the first argument, because obtaining evidence as to self-identity’s potential explanation of intention first requires resolving the issues regarding operationalization. Currently, self-identity items used in studies are not standardized; each study can use their own interpretation and create their own items. If there is no standardization of measurement, then there is no unity of the construct, hindering investigation of potential overlap with other determinants or the effect of past behavior. Self-identity currently faces these operationalization issues because of its history, a brief overview of which is outlined below.

### Overview of self-identity as a fourth variable in RAA

Self-identity was first proposed as an additional construct by Charng, Piliavin, and Callero (1988) in the context of blood donation. It was not the first study to incorporate self-identity in the prediction of intention, but it was the first to specifically do this in the context of a theory in the Reasoned Action Approach lineage, specifically its first incarnation as the Theory of Reasoned Action (TRA). The argument that Charng et al. (1988) made to include self-identity was that repeated behaviors are often part of an identifiable role. Using principal component analysis, the distinction between self-identity, attitude and subjective norm was demonstrated. The self-identity items had high loadings on the first component, ‘role-person merger’, and low loadings on the second component, where the attitude items had high loadings. Charng et al. (1988) found comparable results for subjective norm and inferred that these self-identity items measured a different underlying construct (the attitude and subjective norm items exhibited the reverse pattern). In addition, they found that the proportion of explained variance of intention increased with 7% when self-identity was added to the model, suggesting that self-identity could be a useful addition to the TRA. Their study had one important limitation: the comparison between TRA and self-identity was not planned beforehand, resulting in TRA measurements that where not consistent with the TRA specification. Thus, the increase of 7% of self-identity could be explained by not measuring the TRA constructs correctly. If that would be the case, then self-identity would not be a useful addition to the model because those 7% explained variance belonged to the measured TRA constructs.

Sparks and Shepherd (1992) assumed the same logic: the increased explained variance of intention resulted from not measuring the TRA constructs correctly instead of self-identity having an added value in and of itself. They hypothesized that if attitude were operationalized conform the TPB guidelines, self-identity would no longer contribute to the prediction of intention. Their results were contrary to their expectations: there was an increase of 4% when self-identity was included in the prediction of intention. Despite this finding, Sparks and Shepherd (1992) remained sceptical and argued that a better measure of attitude, including moral and affective components, would explain the significant effect of self-identity.

This hypothesis drove most research done since then, until a review showed that over six studies, self-identity accounted on average for 1% of the explained variance in intention above and beyond the conventional TPB variables (Conner & Armitage, 1998). Conner and Armitage pointed out that self-identity could be an important determinant of intention in certain behaviors and argued that future research should look at the processes of how self-identity influences intention, rather than merely checking whether self-identity adds variance to the prediction of intention.

Terry, Hogg, and White (1999) tried to delineate self-identity in relation to the TPB variables, past behavior, and social identity. Their measure of self-identity included items of Charng et al. (1988) and Sparks and Shepherd (1992). Principal component analyses distinguished self-identity from the other TPB variables and social identity. Both identity constructs were independent (i.e. their items loaded on different components) and had independent effects in the regressions. These results are in line with previous research: there was an independent contribution of self-identity to the prediction of intention, after controlling for the TPB variables and past behavior. Terry et al. make a plea for more research towards examining the role of identity-related constructs in attitude-behavior relations.

Sparks (2000) wrote about the attitude-behavior relationship and the role of self-identity. Sparks acknowledged that self-identity adds to the prediction of intention, but also identified several problems, especially relating to the operationalization of the construct, which might instead measure attitude, past behavior or even intention. The construct and the possible processes affecting intentions are also discussed. Sparks speculated that self-identity might capture long term interests while the attitude scale might reflect short term goals. As such, self-identity may guide everyday behavior in the face of competing interests or wishes. Furthermore, Sparks suggested that people’s self-identities (regardless of whether they are personal or social) embody a subset of values or interests that are not measured in the attitude scale. In sum, Sparks argued that self-identity remains a different scale of attitude instead of a new determinant. Rise et al. (2010) tested this hypothesis using a meta-analysis and found that self-identity added 6% to the prediction of intention. The independent effect remained and the total additional explained variance increased to 9%, when controlling for the RAA variables and past behavior. Rise et al. (2010) concluded that there is no overlap between attitude and self-identity and gave two arguments for their reasoning. First, the correlation of attitude and self-identity was modest (.14; this corresponds to 2% of explained variance). Second, self-identity had a strong independent effect on intentions when controlling for attitude. These results render it implausible that self-identity and attitude are the same construct. The same argument was made for subjective norms, group identity and past behavior.

### Operational issues of self-identity measurement

Closer examination of the self-identity operationalizations employed in these studies confirms Fishbein and Ajzen’s observation: these operationalizations exhibit considerable heterogeneity, and conceptual independence has not been established properly. For example, principal component analysis was usually used to differentiate self-identity from the other RAA variables (e.g. Charng et al., 1988; Terry et al., 1999). However, the measurement model underlying principal component analysis assumes reflective, rather than latent, constructs (i.e. components reflect every item’s unique variance as well as covariance; e.g. Borsboom, 2006). Factor analysis does assume underlying latent constructs (and therefore allows for measurement error in the items) but was only used in one very recent study (Reid, Sparks, & Jessop, 2018). In combination with the heterogeneity in self-identity operationalizations and the fact that each study only explored self-identity’s role with respect to a single behavior and a single population, it renders the existing evidence base too tenuous to draw conclusions as to whether self-identity is conceptually independent from the conventional RAA constructs (hereafter, we will use ‘RAA’ to refer to all theories in the RAA lineage). Moving forward requires uniformity in the measurement of self-identity. Uniformity in measurement of a construct starts with uniformity in definition. As is the case with operationalizations, several definitions of self-identity in an RAA context have been proposed.

### Defining self-identity

The definitions that have been put forward converge in some respect while diverging in others. Three definitions that appeared most influential in self-identity operationalization will be discussed. First, the definition of Charng et al. (1988), as it is one of the first definitions that was provided. Second, the definition of Conner and Armitage (1998), as many of the consecutive studies based their definition on this paper. Third, the definition of Rise et al. (2010), because of this meta-analysis’ role in Ajzen and Fishbein’s discussion of self-identity. Together, the three definitions illustrate how the definition of self-identity has changed over thirty years of research.

Charng et al. define self-identity as a role identity: ‘[…] a set of characteristics or expectations that simultaneously is defined by a social position in the community and becomes a dimension of the actor’s self […]’ (1988, p. 304). This definition consists of three elements. First, self-identity is a set of characteristics or expectations. Second, those characteristics relate to the social position in the community. Third, they relate to the actor’s self.

Conner and Armitage define self-identity as ‘[…] the salient part of an actor’s self which relates to a particular behavior. It reflects the extent to which an actor sees him- or herself as fulfilling the criteria for any societal role […]’ (1998, p. 1444). This consists of four elements. The first element establishes self-identity as a *salient* part of the self. The second element establishes that this part has to relate to a specific behavior. The third element describes self-identity as how people view themselves. The fourth element constrains this viewing of oneself to a societal role.

Rise et al. (2010) define self-identity as applied to smoking, and describe it as a ‘me-identification’. They define this as ‘identification of the self as a smoker and include[ing] the meanings, expectations, and activities related to being a smoker’ (2010, p. 1099). This definition contains two elements. First, the identification of the self with a specific behavior (e.g. smoking). Second, the meaning, expectations and activities relating to the behavior are included in this definition.

There are three problems with this definition. First, a key in these definitions is the identification with the behavior – but for the definition to be useful, the term ‘identification’ first requires exact definition itself. A second problem in some of these definitions is the requirement of self-identity to be a form of social identity. This is problematic for two reasons: (1) requiring social construal guarantees confounding (conceptual dependence) with RAA’s perceived norm construct; and (2) requiring a societal role precludes including self-identity as a generic RAA variable, because for many behaviors, no relevant societal roles exist, while identifying with the behavior is still possible. For example, equivalents to being ‘a teacher’ or ‘a mother’ may not exist for people who always have milk in their tea (e.g. there is no such societal role as ‘a tea-milker’), yet these people may still identify with this behavior. If a construct is to be included in a future RAA iteration, it should conform to the same imperative of wide applicability as the other RAA constructs. The third problem concerns the definition of Rise et al. (2010): not all behaviors have the convenience of relating to a one-word identity such as ‘smoker’. Therefore, the existence of such a clearly delineated and labeled identity should not be a prerequisite in the definition of self-identity.

We therefore propose the following definition for self-identity as a construct that may be studied in the context of the RAA. People form representations of the world, and some of those representations pertain to the self. This set of attributes together form one’s general self-identity (i.e. unrelated to specific behaviors). However, most of these attributes are relatively trivial (e.g. all humans have a brain, but brains are usually not part of their self-identity). How central a given attribute is to one’s self-identity in general (its centrality) can depend on the degree to which it is perceived to distinguish one from others, confers positive characteristics, or is instrumental. For example, health is seen as desirable, so being ‘a healthy eater’ is desirable. Similarly, if sophistication is seen as desirable, and having well-developed tastes are seen as indicative of sophistication, then being somebody who is very picky about one’s coffee blends and preparation methods can be seen as desirable. As a final example of an instrumental function of self-identity, being ‘an addict’ can be used to excuse relapse.

Also, a given attribute can be more or less related to a given target behavior (e.g. being a ‘vegan’ is intrinsically related to eating behaviors). Given that one’s self-identity is defined as all these attributes that pertain to one’s self (weighted by their centrality), it follows that for different people, a given target behavior can be differentially relevant to their self-identity. In terms of operationalizations, this implies that this relevance of a behavior to one’s self-identity should be what is measured (note that this is not the same as general relevance of a behavior).

### Operationalization of self-identity

The aim of the present review is to identify all core aspects of/self-identity that are captured in the items derived from previous research (in the context of the RAA and its predecessors) and create a standardized scale of self-identity items in congruence with our redefined definition. These core aspects are reformulated in prototypical items that are operationalized using the guideline from Ajzen (2002). In the next paragraphs the guideline for RAA operationalization are outlined briefly and linked to our selection of prototypical items.

A central principle in the operationalization of RAA constructs is the requirement to realize compatibility in terms of their target, action, context, and time (TACT; Ajzen, 2002). For example, when measuring intention to go for a run for at least twenty minutes somewhere in the next month, when measuring expectations and evaluations (as subconstructs of attitude), these should also relate to going for a run for at least twenty minutes somewhere in the next month. Not only is people’s intention to go for a run for at least four hours tonight at 3:00 typically markedly different, the RAA holds that this intention is not (necessarily) predicted by attitude regarding going for a run for at least twenty minutes somewhere in the next month. This is an example of violating the construct’s operationalisations’ compatibility in terms of TACT. In a way, these four dimensions (target, action, context, time) are arbitrary; any behavior can be specified in terms of a specific intersection of a large number of dimensions (Peters & Crutzen, 2017). One dimension that Ajzen explicitly discusses is specificity: intention to engage in some form of exercise in the next month is not the same as intention to engage in running in the next month. This ability to capture this breadth of variation in potential target behaviors is a central principle in the RAA that additional determinants must adhere to. This means that self-identity items such as ‘I am a smoker’, even in their template form (‘I am a […]’), do not lend themselves as operationalisations of additional RAA constructs: they do not enable achieving compatibility in terms of TACT or specificity.

It should be noted that the measurement of RAA has been criticized (see Sniehotta, Presseau, & Araújo-Soares, 2014) for its limited predictive validity. According to Sniehotta et al., the sufficiency assumption would be empirically and conceptually indefensible as age, socio-economic status and other variables such as identity have an effect beyond the meditation of RAA variables. Ajzen (2015) and Conner (2015) both argued that Sniehotta et al. (2014) lacked evidence to support this statement and have misinterpreted certain elements of the theory. For example, age and socio-economic status are already in the model (see Fishbein and Ajzen, 2010, p. 22). The argument about additional predictors could also be accounted for by imperfection of RAA measurement, such was the case in Ajzen and Sheikh study (2013) about anticipated regret. The criticism of Sniehotta et al. (2014) does not provide a solid foundation to reject how RAA determinants are measured and thus the principles as described by Ajzen (2002) are used to operationalize the prototypical items.

To develop an operationalization for self-identity that does not suffer from the problems described above, we synthesized all self-identity operationalizations as employed in self-identity research in the context of the RAA (and its predecessors). In this synthesis, we applied the definitions and operational constraints we just outlined to obtain a final set of coherent items that are compatible with the RAA.

## Method

### Search strategy

Based on the literature available to us at that point, we formulated a query that combined two concepts, self-identity and the Reasoned Action Approach, and contained the terms plausibly used to refer to these concepts in articles’ titles and abstracts. Specifically, the following query was submitted to the PsycINFO database:

((AB ((‘role identity’ OR ‘self-identity’ OR ‘selfidentity’ OR ‘self identity’))) OR (TI ((‘role identity’ OR ‘self-identity’ OR ‘selfidentity’ OR ‘self identity’)))) AND ((TI ((theory of reasoned action) OR (theory of planned behavior) OR (theory of reasoned action) OR (tra) OR (tpb) OR (raa))) OR (AB ((theory of reasoned action) OR (theory of planned behavior) OR (theory of reasoned action) OR (tra) OR (tpb) OR (raa))))

The query searched in the abstract and title and resulted in 117 articles. We then first applied the descendency approach to these 117 articles. To this end, each of the 117 articles was entered into Google Scholar, and we then searched all articles that cited each of these 117 studies with the following query:

allintitle: ‘self-identity’ OR ‘selfidentity’ OR identity OR ‘theory of reasoned action’ OR ‘theory of planned behavior’ OR ‘theory of reasoned action’ OR tra OR tpb OR raa

### Screening

**Figure 1.**
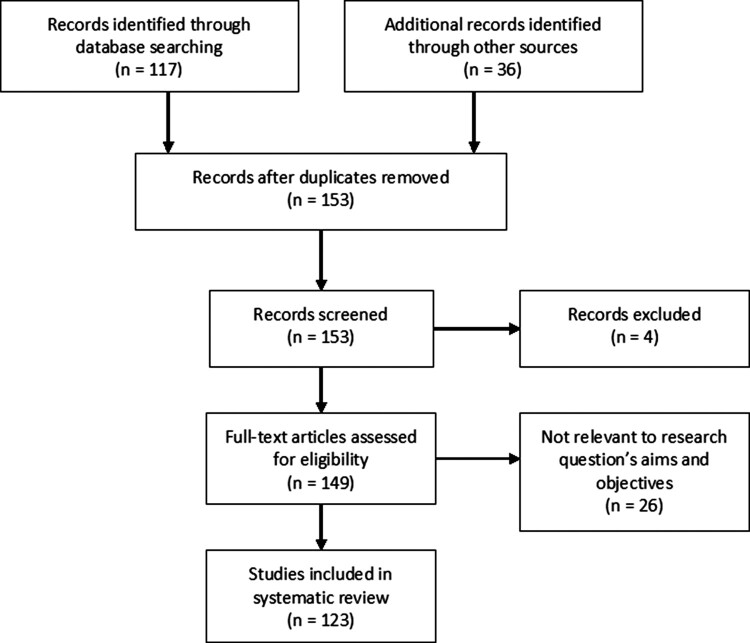
PRISMA flowchart illustrating the search strategy.

The 117 articles identified in the PsycINFO query as well as the 36 additional articles identified through the descendancy approach were screened by MS by applying the following criteria. First, studies that measured the self-identity – intention relationship without specifically mentioning the RAA were excluded, such as Biddle, Bank, and Slavings (1987). Second, studies into constructs that were related to, but different from, self-identity were excluded (e.g. Fitzmaurice, 2005; Case, Sparks, & Pavey, 2016). Studies that incorporated both social identity and self-identity were not excluded (e.g. Terry & Hogg, 2016). Third, studies that were not empirical and quantitative were excluded. Fourth, studies that did not list one or more self-identity items were excluded (e.g. Bozionelos & Bennett, 1999). When it was unclear whether a study met one or more criteria, all authors discussed and resolved these cases. Using these criteria, 30 of the 153 articles were excluded (123 were retained). The search strategy is illustrated using a PRISMA flowchart (from Moher, Liberati, Tetzlaff, & Altman, 2009) in Figure 1. The operationalizations extracted from these studies then went through the categorization process.

### Full disclosure

We fully disclosed all materials for this study at the Open Science Framework at https://osf.io/whef9/. This includes the BibTeX databases used for screening and item extraction as well as the R scripts used to conduct the analyses.

## Results

### The categorization process

All self-identity operationalization items as used in the included studies were extracted to a BibTeX database, which was imported into R. Closer inspection revealed that very few studies used the same items. This was partly due to heterogeneity in studies’ target behaviors, but also reflected rephrasing of items in different studies.

To make a comparison possible between all items, we first reduced the list of items to their core aspects by looking for central phrases. For example, ‘I am the kind of person that exercises regularly’ and ‘I am the kind of person that uses condoms’ are different questions, but essentially they share the same fundamental core as question, namely ‘I am the kind of person that […]’. To render this process transparent and reproducible, regular expressions were used to collapse the questions to sets of such core questions. Regular expressions are symbolic representations of text strings that facilitate pattern matching, allowing, for example, formulation of an efficient expression that matches all valid email addresses.

We started with a list that contained all items of all included papers. Looking at the list we would try to find the keywords that captured each question’s core aspect and formulated this in a regular expression that would match similar all similar items. For example, the core aspect in the question ‘I am a kind of person who is a donor’ is the expression of being a certain ‘kind of person’. The regular expression ‘kind of person’ matches all text strings containing exactly that substring. All such items were therefore placed into that category and removed from the list. Another example is ‘I am concerned about exercising regularly’, where the core aspect expresses concern about performing a behavior. This is captured in the regular expression ‘concerned about’, causing all other questions matching that substring to be categorized into that category. The regular expressions in the list were applied sequentially, meaning that we placed more specific regular expressions before generic expressions that would match many questions.

**Figure 2.**
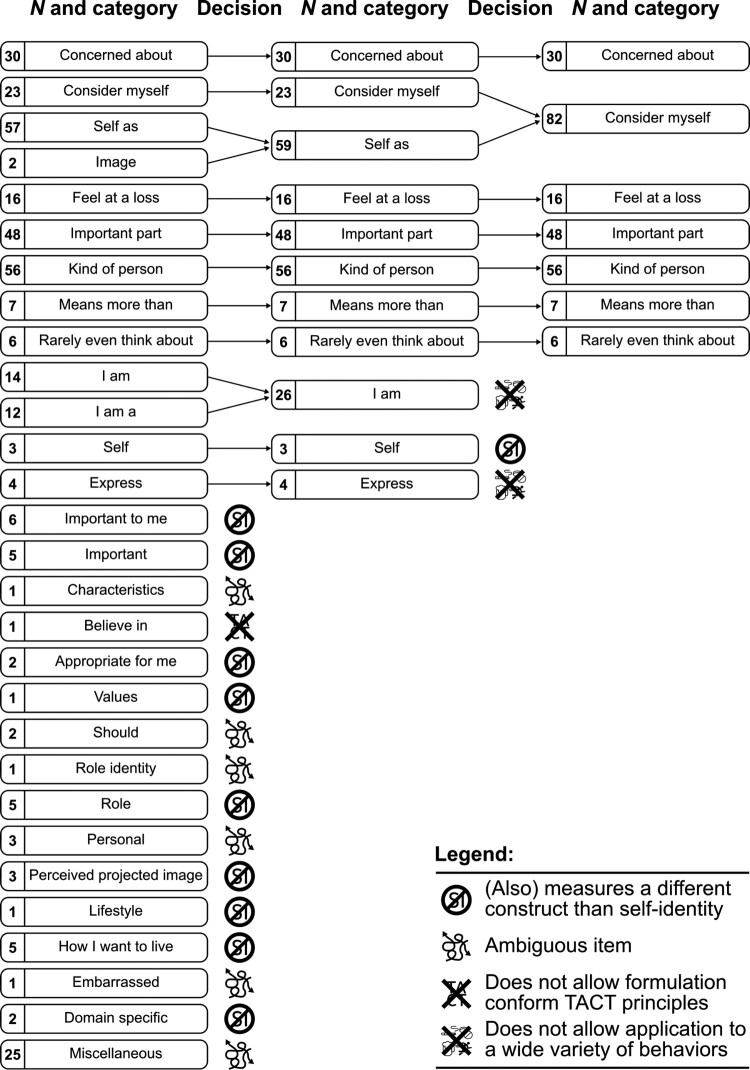
The selection process of prototypical items.

We identified 29 core aspects that were measured using different central phrases. The initial core aspects are displayed in the left-most column of Figure 2. The number before each category lists the number of items in that core aspect.

After identifying the 29 categories, we then closely inspected these categories with two aims: first, to detect any incorrectly categorized items (in which case the relevant category’s regular expression was adjusted to correct this, by including additional characters, thereby making it more specific and preventing it from matching the items it should not match); second, to merge categories that contained very similar items. We applied this procedure iteratively until we had reached homogeneous categories.

### The process of extracting prototypical items

Having reached homogeneous categories, we started category selection to ultimately enable formulation pf prototypical items. In this process, elimination of an entire category could be based on one of four reasons (see the supplemental files for more details). First, some categories captured items that operationalized a different construct than self-identity. The central phrase ‘appropriate for me’ is not congruent with measuring the relevance to one’s self-identity as defined in the introduction and thus is a different construct. Second, the items in some categories did not explicitly refer to the target behavior (and so were inconsistent with TACT). Third, the items in some categories operationalized ambiguous (sub-)constructs, such as items in category ‘Personal’, which could be interpreted in a variety of ways and were therefore too general. Fourth, some categories contained items that were phrased in a way that precluded application to a wide variety of behaviors, such as items in the category ‘I am a’ (e.g. ‘I am a smoker’) and thus be too specific. In total, 71 items have been excluded from the 342 items.

In addition to eliminating some categories, we merged others if they contained items measuring the same underlying construct as can be seen in the middle and right-most columns in Figure 2. A clear example of merging are the two categories ‘I am’ and ‘I am a’, which differed in that items in the ‘I am’-category used more flexible identities, whereas items in the ‘I am a’-category concerned named roles (the use of named roles prohibits application of a question to a wide variety of behaviors, and adjusting the question to correct this would reformulate the items to the ‘I am’ variety). The category ‘Image’ was also a category that could be merged with another category, as ‘image’ can be defined as a general impression which essentially falls in the same category as ‘Self as’, which also contains ‘see myself as’ items. After eliminating and merging, only seven categories remained. In the final step, we identified the prototypical item representing each of the remaining categories. A prototypical item from each ultimate category is listed in Table 1. These items correspond to the right-most column in Figure 2. Note that the frequency of occurrence of each of these items in the literature cannot be taken as evidence of their importance or relevance to self-identity. There is no reason to assume that when researchers select items to use in a study, there is a process at work that causes researchers to converge on (and therefore more frequently use) items that are more valid or reliable. Such selection and ranking of items would require dedicated research: in general, how often something occurs in the literature is not a good indicator of anything except of frequency of occurrence in the literature.

**Table 1.** The prototypical items of self-identity.

Items measuring self-identity		
**I see myself as someone who is concerned about [target behavior]**	Disagree	Agree
**I see myself as someone who [target behavior]**	Disagree	Agree
**I would feel at a loss if I were forced to give up [target behavior]**	Disagree	Agree
**[Target behavior] is an important part of who I am**	Disagree	Agree
**I am the kind of person who [target behavior]**	Disagree	Agree
**For me, [target behavior] means more than just the act itself**	Disagree	Agree
**[Target behavior] is something I rarely even think about**	Disagree	Agree

## Discussion

Self-identity was frequently proposed as an extension to the RAA, and its inclusion has consistently added explained variance to the prediction of intention within the RAA. Still, Fishbein and Ajzen (2010) decided against inclusion of self-identity as a fourth variable. One of the reasons to exclude self-identity were the operational and conceptual issues surrounding the variable. According to Fishbein and Ajzen (2010) self-identity measures other RAA constructs, such as attitude, perceived norm, past behavior and current behavior.

As this review showed, Fishbein and Ajzen’s observation that self-identity operationalization has so far been exceptionally heterogeneous is accurate. This means that before self-identity can be seriously evaluated as potential addition to the RAA, these operational and conceptual issues beg resolution. The present review sought to do exactly this: compile a list of all items that have been used to measure self-identity in an RAA context so far, and then compile this list into a short list of prototypical items that can then be used going forward, for example, in studying the potential overlap with other determinants.

Using our integrated definition of self-identity, as well as the RAA-related operationalization principles (that items should be applicable to a wide range of behaviors and it should be possible to formulate them using the TACT and compatibility principles), 342 items from 123 studies where identified and categorized. All items measure aspects of self-identity, except for three items that directly asked participants whether as behavior fit with or was central to their self-concept. Such a direct approach may yield a very economic measure of self-identity, but it may also be too prone to social desirability or other biases. Given this exceptional status, these items were excluded in the preliminary list. The resulting list of seven prototypical self-identity items is shown in Table 1. These items lend themselves well for further psychometric study.

The present systematic review of the operationalization of self-identity has several limitations. First, our query for finding relevant articles could have been broader. For example, we later identified several self-identity studies that our search strategy failed to locate, such as Biddle et al.’ (1987) study, as the title and abstract did not refer to the RAA or one of its predecessors. If we would have added, for example, the term ‘intention’ to the query, we would have identified more studies and studies such as Biddle et al. (1987) could have been incorporated. However, we do not expect that this lacuna resulted in a systematic bias in the final list of included studies, and given the degree of overlap in items in the studies we did include, it is unlikely we missed additional operationalizations.

A second limitation is the strict adherence to the criteria we formulated as requirements for operationalizations in the context of the RAA: had we not excluded operationalizations that could not be applied to a wide variety of behaviors, or items that did not lend themselves to formulations according to the TACT principle, we might have arrived at a different item set. In other words, it is important to realize that the operationalization of self-identity that we compiled is very much optimized for use as an RAA extension. This may mean that some aspects of self-identity that are incompatible with the RAA framework, or with the TACT principle, were omitted.

A main strength of this review is that this approach did enable proposing a list of prototypical items that lends itself well to further study. This can resolve some of the limitations we listed above. A second strength is the completely fully disclosed process: this enables other researchers to closely scrutinize all our decisions, as well as take this work and possibly come to different decisions.

Future research can move forward by using the prototypical items to assess the conceptual independence of self-identity within the RAA. Inspection of correlation matrices and employing exploratory or confirmatory factor analysis can then guide further research. The study of Reid et al. (2018) is a good example of how the debate surrounding self-identity can be developed further using confirmatory factor analysis. Their results suggested that the importance scale and self-identity are not the same construct, but this suggestion was based on one sample and one behavior. It is important that future research explores self-identity’s potential conceptual independence over multiple behaviors and populations. Such studies may lead to improvements of the initial pool of seven prototypical items as presented in this synthesis, eventually arriving at a set of items that lends itself well for measuring self-identity over a wide range of behaviors and populations.

If an operationalization of self-identity emerges that is consistently conceptually independent from the RAA constructs, then the next step would be to test whether self-identity, in that operationalization, has added value over and above those RAA constructs. If that is the case, it is important to replicate that result, and if it proves robust, to abandon the discussion as to whether self-identity has an added value and instead accept that it does, and shift attention and resources to behavior change principles (Crutzen & Peters, 2018) that can be used to target self-identity.

If, however, no conceptually independent operationalization of self-identity can be arrived at, or if a conceptually independent operationalization can be established but it does not explain aspects of intention that remain unexplained by attitude, perceived norm, and perceived behavioral control constructs, then this discussion can be abandoned as well. Whatever the outcome, we hope that the prototypical self-identity items we presented herein can help resolve this matter.

It is important to emphasize that whether self-identity has a role as an RAA extension is unrelated to its value as a behavioral determinant. If consistent (i.e. uniform) operationalization allows explanation of either intention of behavior, and if behavior change principles are available or can be developed that successfully shift people’s self-identity away from unhealthy or harmful behaviors, thereby improving their health or quality of life, that suffices to render self-identity a useful construct in behavior change research. The prototypical list of items presented in this synthesis, therefore, may prove useful regardless of whether self-identity should be included in a future RAA iteration or not.
